# Crystallization-Induced Gelling as a Method to 4D Print Low-Water-Content Non-isocyanate Polyurethane Hydrogels

**DOI:** 10.1021/acs.chemmater.1c00913

**Published:** 2021-09-14

**Authors:** Noé Fanjul-Mosteirín, Robert Aguirresarobe, Naroa Sadaba, Aitor Larrañaga, Edurne Marin, Jaime Martin, Nicolas Ramos-Gomez, Maria C. Arno, Haritz Sardon, Andrew P. Dove

**Affiliations:** † School of Chemistry, University of Birmingham, Edgbaston, Birmingham B15 2TT, U.K.; ‡ Department of Chemistry, University of Warwick, Gibbet Hill Road, Coventry CV4 7AL, U.K.; § POLYMAT, University of the Basque Country UPV/EHU, Joxe Mari Korta Center, Avda Tolosa 72, 20018 Donostia-San Sebastian, Spain; ∥ Department of Mining−Metallurgy Engineering and Materials Science, POLYMAT, School of Engineering, University of the Basque Country UPV/EHU, Alameda de Urquijo s/n, 48013 Bilbao, Spain; ⊥ Grupo de Polímeros, Departamento de Física e Ciencias da Terra, Centro de Investigacións Tecnolóxicas (CIT), Universidade da Coruña, Esteiro, Ferrol, 15471 Spain

## Abstract

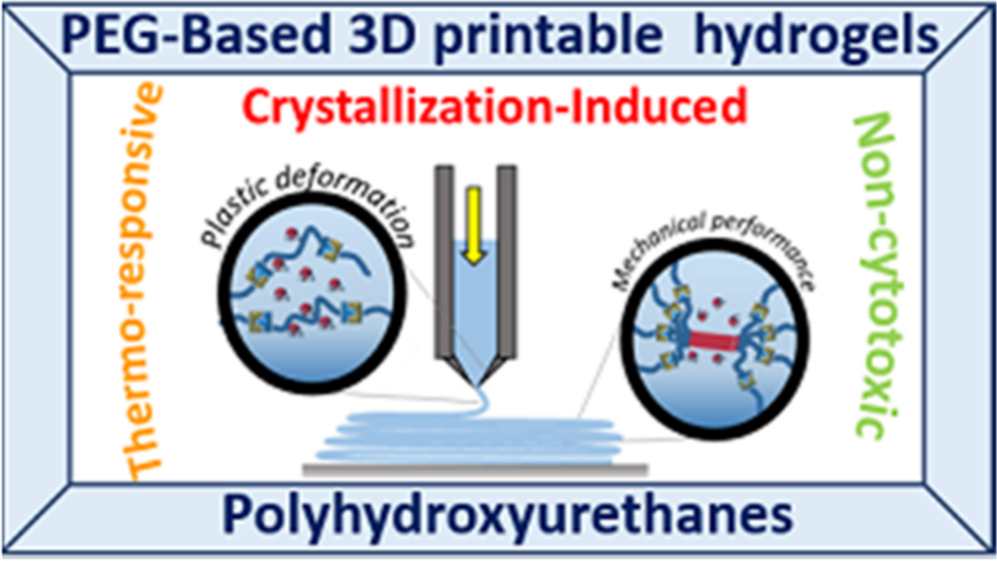

The use of three-dimensional (3D) printable hydrogels for biomedical applications has attracted considerable attention as a consequence of the ability to precisely define the morphology of the printed object, allowing patients’ needs to be targeted. However, the majority of hydrogels do not possess suitable mechanical properties to fulfill an adequate rheological profile for printability, and hence, 3D printing of cross-linked networks is challenging and normally requires postprinting modifications to obtain the desired scaffolds. In this work, we took advantage of the crystallization process of poly(ethylene glycol) to print non-isocyanate poly(hydroxyurethane) hydrogels with tunable mechanical properties. As a consequence of the crystallization process, the hydrogel modulus can be tuned up to 3 orders of magnitude upon heating up to 40 °C, offering an interesting strategy to directly 3D-print hydrogels without the need of postprinting cross-linking. Moreover, the absence of any toxicity makes these materials ideal candidates for biomedical applications.

## Introduction

Three-dimensional (3D) printing has emerged as an attractive technique to readily fabricate biomedical devices, as a consequence of its inherent process flexibility, the potential to introduce bioactive functionalities, and the advantage to customize the printed material to the patient’s needs.^1−3^ Among the wide variety of scaffolds designed for tissue engineering applications, hydrogels represent the ideal candidates to replace soft tissues, owing to their high water content, cytocompatibility, and tunable mechanical properties. Indeed, hydrogels for 3D printing have been the center of fundamental as well as industrially relevant research.^4^ However, hydrogels need to be formulated specifically for each bioprinting technology to fulfill the processing requirements of each technique.

To date, a number of bioprinting methods have been explored, including inkjet printing, stereolithography, and extrusion-based printing.^5,6^ Among them, extrusion-based printing represents one of the most efficient techniques for bioprinting, as it does not require photocuring, which could have a negative impact on cell viability.^7^ Despite the doubtless benefits of hydrogels as cell-laden materials, their preparation by 3D printing has been limited,^1,5,8^ mainly owing to the lack of materials with adequate rheological and mechanical properties that allow precise control over the printing process.^9,10^ Therefore, to meet both criteria, hydrogels must be modified after printing to reach suitable mechanical properties.

Shear-thinning hydrogels prepared by noncovalent interactions are excellent candidates for 3D bioprinting as a consequence of their ability to flow during the printing process and rapidly undergo a sol–gel transition after printing—a consequence of the noncovalent interactions re-forming.^11^ Such printable hydrogels have been formulated based on weak interactions, such as hydrogen bonds, host–guest interactions, and metal–ion interactions among others.^12^ Their non-Newtonian behavior and thixotropic properties allow 3D scaffolds with high shape fidelity and good mechanical properties to be obtained.^4,12^ However, they often require postprinting cross-linking to achieve the desired mechanical performance, which may lead to undesirable cell death.^13^

In our search for a novel methodology for cytocompatible hydrogel inks for 3D printing that displayed good mechanical performance, special attention was directed to semicrystalline hydrogels with transition temperatures close to body temperature. While most of synthetic hydrogels are amorphous, semicrystalline hydrogels possess ordered aggregates contributing significantly to their mechanical performance. Indeed, these hydrogels can undergo an abrupt and reversible change from a solid-like to a liquid-like state at the melting temperature, opening up new opportunities for four-dimensional (4D) printing.^14−16^ We hypothesized that, above the melting temperature, the hydrogel could behave as a fluid, facilitating the printing process, while below the melting temperature, it could form a gel, achieving the desired mechanical performance. Inspired by this concept, we designed semicrystalline hydrogels using poly(ethylene glycol) (PEG)-based polyurethanes (PUs) that displayed both good mechanical properties at room temperature (rt) as well as the ability to flow during the printing process.^17^ PUs have been considered as one of the most versatile class of materials owing to their ability to phase-separate into hard and soft segments that is responsible for their excellent mechanical stress and elasticity.^18^ In turn, PUs have been used to fabricate heart valves, vascular grafts, catheters, and prostheses, owing to their versatility and biocompatibility.^19^

The use of PEG as a soft segment has been shown to be critical to produce semicrystalline polyurethanes.^20^ In a previous report, we prepared polyurethanes with tunable melting temperatures from the step-growth polycondensation of pentafluorophenyl-activated carbonates and diamines close to body temperatures.^21^ However, while polyurethanes could be obtained in aqueous media owing to the high reactivity of the activated carbonates, toxic pentafluorophenol is generated as a side product from this reaction. To overcome this limitation, Detrembleur *et al.* reported the synthesis of poly(hydroxyurethane) (PHU) hydrogels, taking advantage of the reaction between five-membered bis-cyclic carbonates (5MCC) and tris-amines without generating any side product.^22^ However, as a consequence of the low reactivity of five-membered bis-cyclic carbonates, gelation was performed at 60 °C and in the presence of organic solvents, which can eventually prevent their use in the biomedical field. Further research from the same group synthesized hydrogels at room temperature using five-membered bis-cyclic carbonates. In addition, the aminolysis of the five-membered bis-cyclic carbonates in water is strongly dependent on the p*K*_a_ of the amine used and the CO_2_ release is difficult to prevent, in turn making it difficult to control the structure of the hydrogel.^23,24^

Herein, semicrystalline hydrogels have been prepared using highly reactive N-substituted eight-membered bis-cyclic carbonates (8MCC), PEG diamine, and multifunctional tris(2-aminoethyl)amine (TAEA). The use of this cyclic carbonate provides high reactivity and excludes the formation of any condensate during the polymerization, while the use of PEG will introduce semicrystalline domains into the hydrogel. We show that the crystallization-induced gelation provides adequate rheological and mechanical properties that allow the 3D printing of pre-formed biocompatible hydrogels with suitable mechanical properties, thus avoiding the use of postfunctionalization treatment. This work could open new opportunities for the printing of hydrogels for tissue engineering.

## Experimental Section

### Materials and Methods

All commercially available starting materials were purchased from Sigma-Aldrich, Alfa Aesar, or Acros Organics and used as received unless otherwise indicated. Tris(2-aminoethyl)amine was stored in a glovebox as it is moisture-sensitive. NMR spectra were recorded on a Bruker Avance 300 MHz spectrometer or a Bruker Avance III HD 300 MHz spectrometer. Chemical shifts are reported in parts per million (ppm) and referenced to the residual solvent signal (dimethyl sulfoxide (DMSO): ^1^H, δ = 2.50 ppm, ^13^C, δ = 39.5 ppm, tetramethylsilane (TMS): ^1^H, δ = 0.00 ppm). Multiplicities are reported as s = singlet, brs = broad singlet, d = doublet, dd = doublet of doublets, dt = doublet of triplets, ddd = doublet of doublet of doublets, ddt = doublet of doublet of triplets, t = triplet, tt = triplet of triplets q = quartet, quint = quintet, m = multiplet. Multiplicity is followed by coupling constant (*J*) in hertz and integration. Rheological testing was carried out using an Anton Paar MCR 302 rheometer equipped with a parallel-plate configuration with a diameter of 25 mm, under linear viscoelastic conditions. A Peltier system was used to control the temperature throughout the study. Data were analyzed using RheoCompass software. Dynamic mechanical thermal analysis (DMTA) measurements were conducted in compression mode in a dynamic mechanical analyzer, Triton 2000 DMA (Triton Technology). Prismatic samples were heated from −20 to 80 °C at a constant heating rate of 4 °C/min and a frequency of 1.0 Hz. Additionally, frequency sweeps were conducted in the range of 0.1–35 Hz at a constant temperature of 25 °C. The compression ratio was fixed at 1.5. The crystallization process of the samples was measured by differential scanning calorimetry (DSC) in a Pyris 1 DSC (PerkinElmer) equipped with an intracooler, with heating and cooling rates of 10 °C/min. Samples were prepared using standard aluminum capsules. Grazing-incidence wide-angle X-ray scattering (GIWAXS) measurements were performed at the BL11 NCD-SWEET beamline at ALBA Synchrotron Radiation Facility (Spain). The incident X-ray beam energy was set to 12.4 keV, and the incident angle employed was between 0.2°. The exposure time was 1 s. The scattering patterns were recorded using a Rayonix LX255-HS area detector. Temperature-resolved *in situ* experiments were conducted at a heating rate of 20 °C/min using a customized Linkam THMS 600. The printing process was carried out in a 3D-Bioplotter (Developer Series, EnvisionTEC), and the printing geometries were originally designed in SolidWorks 2016 × 64 Editor. Extrusions were conducted at 25 °C, and the needle diameter used was 0.25 mm. In addition to this, a pre-flow of 0.3 s and a post-flow of 0.1 s were necessary. To assess the cytotoxicity of the developed hydrogels, ISO/EN 10993 procedures were followed. Extracts of test materials were obtained by incubating complete medium (Dulbecco’s modified Eagle’s medium (DMEM) + 10% fetal bovine serum (FBS) + 1% penicillin/streptomycin (P/S)) with the hydrogels (ratio of material surface to extract medium = 3 cm^2^/mL) for 24 h at 37 °C in a humidified atmosphere containing 5% CO_2_. Complete medium and complete medium + 10% DMSO were employed as negative and positive controls, respectively. HeLa cells were seeded at a density of 25 000 cells/well on a 24-well plate in complete medium. After 1 day in culture, complete media was replaced by extracts of test materials or control media (*i.e.*, negative control: DMEM + 10% FBS; positive control: DMEM + 10% FBS + 10% DMSO). Cell viability was then evaluated using the alamarBlue assay after 24 and 72 h. Cell morphology was also analyzed after rhodamine phalloidin and 4′,6-diamidino-2-phenylindole (DAPI) staining at selected time points. For that purpose, the cells were fixed with 4% paraformaldehyde and repeatedly washed with Hank’s balanced salt solution (HBSS). Then, they were permeabilized with 0.5% Triton X-100 in HBSS for 10 min. After that, the cells were incubated in a 1% bovine serum albumin solution in HBSS in the presence of rhodamine phalloidin (0.066 μM) and DAPI (300 nM) for 15 min. The cells were finally washed twice with HBSS-T (0.1% Tween 20) and HBSS prior to their observation in an inverted fluorescence microscope (Nikon Eclipse Ts2). Alternatively, HeLa cells were also seeded in the presence of the hydrogel with the aid of a transwell membrane (poly(ethylene terephthalate) (PET) membrane, diameter: 6.5 mm, thickness: 10 μm, pore size: 8 μm). Briefly, the hydrogel (*ca.* 200 mg) was placed in the upper chamber, together with 100 μL of complete medium, whereas HeLa cells (25 000 cells/well) were seeded in the bottom chamber of 24-well plates. Positive and negative controls were also considered in this experiment. After 1 day of incubation, the metabolic activity of the cells was evaluated as mentioned above.

### Synthesis of 6,6′-(Ethane-1,2-diyl)bis(1,3,6-dioxazocan-2-one) (**1**)

Synthesis of **1** was conducted following a literature procedure.^25^*N*,*N*,*N*′,*N*′-Tetrakis(2-hydroxyethyl)ethylenediamine (5.00 g, 21.16 mmol), 1,8-bis(dimethylamino)naphthalene (2.25 g, 10.48 mmol), and anhydrous tetrahydrofuran (THF) (400 mL) were added to a 1 L round-bottom flask. Bis(pentafluorophenyl)carbonate (18.4 g, 46.69 mmol) in anhydrous THF (50 mL) was then added, and the solution was stirred at room temperature for 2 h. The reaction mixture was then concentrated under reduced pressure, and then treated with an excess of diethyl ether, before being stored in a refrigerator overnight. The precipitated material was then collected and dried to give **1** (4.33 g, 15.02 mmol, yield 70%) as a white solid. Characterizing data was in accordance with that reported previously.^25^^1^H NMR (300 MHz, DMSO): δ 4.08 (t, 8H, ^3^*J*_H–H_ = 5.2 Hz, C*H*_2_–OCOO), 2.76 (t, 8H, ^3^*J*_H–H_ = 5.2 Hz, C*H*_2_–CH_2_–OCOO), 2.62 (bs, 4H, C*H*_2_–N). ^13^C NMR (75 MHz, DMSO): δ 155.5 (OCOO), 69.1 (*C*H_2_–OCOO), 53.6 (*C*H_2_–CH_2_–OCOO), 53.5 (CH_2_–N).

### Synthesis of 4,4′-(Oxybis(methylene))bis(1,3-dioxolan-2-one) (**2**)

Synthesis of **2** was conducted following a literature procedure.^26^ In a 50 mL three-neck round-bottom flask equipped with a magnetic stirrer, a thermometer, and a reflux condenser, diglycerol (a mixture of isomers, 1 g, 6.02 mmol), dimethyl carbonate (3.210 g, 3 mL, 35.6 mmol), and K_2_CO_3_ (5 mg, 0.036 mmol) were added sequentially. The reaction mixture was heated at 70 °C for 24 h. Then, the remaining dimethyl carbonate and methanol were distilled off the mixture at atmospheric pressure at 65 °C over a period of 6 h, before cooling down the reaction mixture to room temperature. The precipitated catalyst was filtered and washed with dimethyl carbonate. The remaining organic phase was evaporated to dryness, and the product recrystallized from ethyl acetate. Bis-glycerol carbonate (**2**) was obtained as a white solid (876 mg, 4.02 mmol, 73% yield). Characterizing data was in accordance with that reported previously.^26^^1^H NMR (300 MHz, DMSO): δ 4.98–4.90 (m, 2H, CH_2_–C*H*–OCOO), 4.52 (t, 2H, ^3^*J*_H–H_ = 8.5 Hz, C*H*^anti^–OCOO), 4.27–4.21 (m, 2H, C*H*^syn^–OCOO) 3.78–3.65 (m, 4H, O–C*H*_2_). ^13^C NMR (75 MHz, DMSO): δ 154.8 (OCOO), 75.4 (CH), 70.4 (*C*H_2_–OCOO), 65.9 (CH_2_–O).

### Synthesis of Poly(hydroxyurethane) Linear Polymers

PEG diamine (100 mg, 0.1 mmol, *M*_w_ = 1000) and bis-cyclic carbonate (0.1 mmol) were dissolved in 1 mL of distilled water. The mixture was stirred overnight and then freeze-dried. Conversion was calculated by ^1^H NMR spectroscopy analysis using relative peak integration values from the reaction crude mixture. The polymerizations were monitored by a diagnostic disappearance of carbonate methylene protons (δ 4.08 ppm, adjacent to the carbonate) and their subsequent reappearance (δ 3.9 ppm, adjacent to the carbamate) (Figure S1).

### Synthesis of Poly(hydroxyurethane) Hydrogels

The general procedure for the preparation of polyhyroxyurethanes with different degrees of cross-linking is summarized in Table S1. Briefly, from a 0.2 M aqueous stock solution of tris-(2-aminoethyl)amine (TAEA) was added to a 5 mL vial, then water was added to a final volume of 400 μL. The appropriate amount of PEG diamine was then added to the mixture and vortexed until complete dissolution. Subsequently, 6,6′-(ethane-1,2-diyl)bis(1,3,6-dioxazocan-2-one) was added to the mixture until it was dissolved (see Table S1). The mixture was left overnight at room temperature without further stirring, and a gel was obtained.

## Results and Discussion

**Figure 1:**
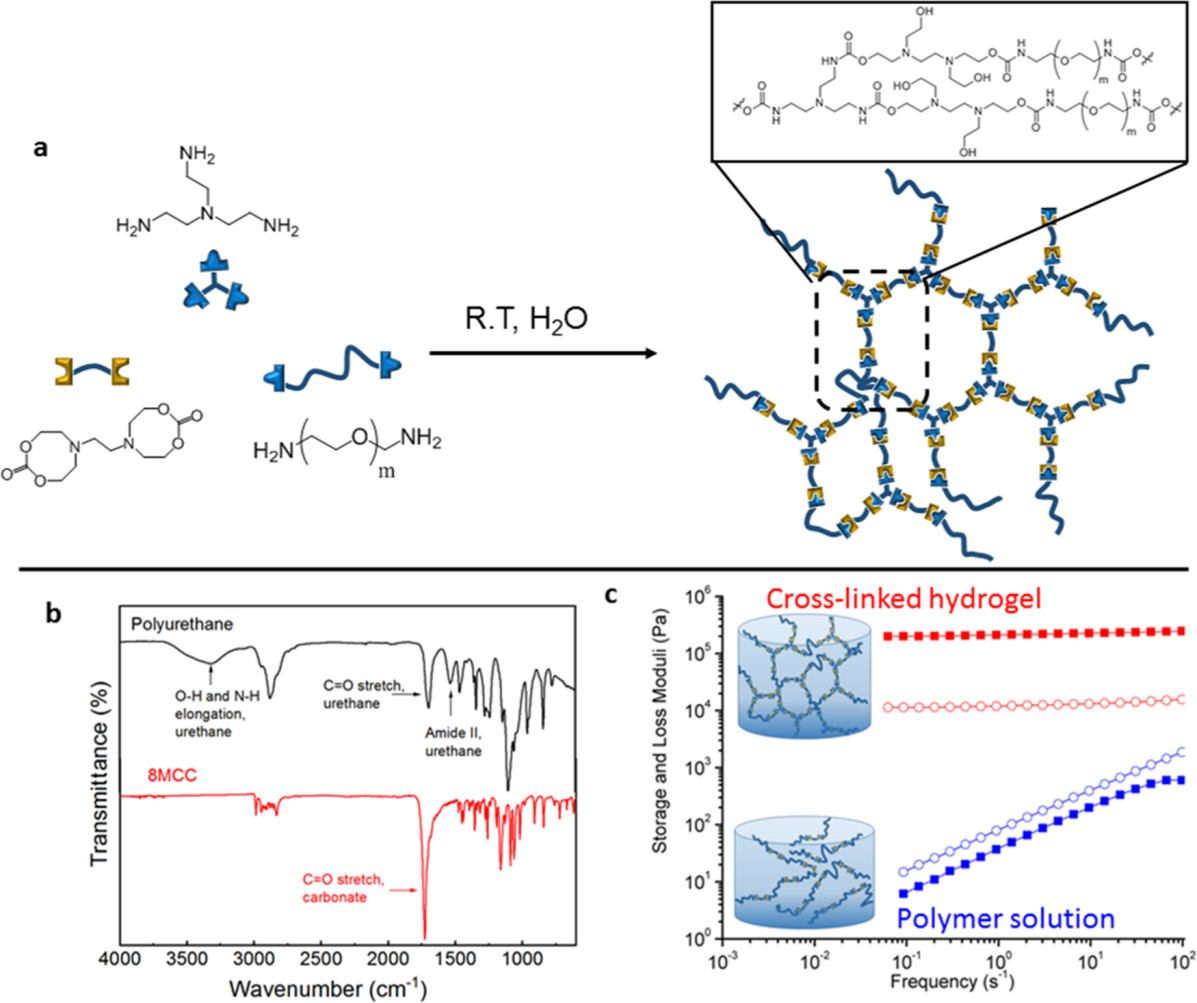
Synthetic pathway used in the synthesis of semicrystalline hydrogels at room temperature (a). Representative FTIR spectra of the synthesized materials (b). Rheological characterization of both materials by frequency sweep experiments; filled symbols correspond to the storage modulus, and empty symbols correspond to loss modulus (c).

The synthesis of poly(hydroxyurethane)s from N-substituted eight-membered bis-cyclic carbonates and diamines was first investigated in water at room temperature, and their reactivity was compared to conventionally used five-membered bis-cyclic carbonates. Model reactions were carried out using linear poly(ethylene oxide) diamine (PEG diamine, *M*_w_ = 1000) with both types of cyclic carbonates (5MCC and 8MCC). ^1^H NMR spectroscopic analysis of the reaction progress revealed that the conversion of eight-membered carbonate-based systems was much higher (91%) than 5MCC (12%) after 24 h at room temperature (Figures S1 and S2). Considering their higher reactivity, 8MCC were explored as a source to prepare hydrogels in aqueous media. Thus, the capability of N-substituted eight-membered bis-cyclic carbonates to form non-isocyanate polyurethane (NIPU) hydrogels was investigated, using PEG diamine *M*_w_ = 3400 as chain extender and tris(2-aminoethylene)amine (TAEA) as a cross-linker (Figure 1a). Monitoring the reaction by Fourier transform infrared spectroscopy (FTIR), it was possible to observe the reduction in the intensity of the carbonate stretching frequency (λ = 1726 cm^–1^) that was completely converted to new urethane carbonyl stretching signals at λ = 1570 and 1720 cm^–1^ (Figure 1b) throughout the hydrogel formation. Additionally, a broad band around λ = 3300 cm^–1^, attributed to the N–H and O–H stretching, was observed. These observations confirm that the reaction between the amine and the 8MCC was efficiently occurring in aqueous media.

To confirm the covalent nature of the formed hydrogels, small-amplitude oscillatory experiments (SAOS) were conducted in frequency sweeps (Figure 1c). Rheological analysis of the materials showed no frequency dependence of the storage (*G*′) and loss (*G*″) moduli, with a predominance of the elastic behavior as expected from a chemically cross-linked network (Figure 1c red). In contrast, an aqueous solution of a linear polymer showed a frequency dependence corresponding to the flow zone (Figure 1c, blue). We subsequently studied the influence of several factors in the gelation process to optimize the formulation to fit with the requirements of the printing process and to obtain materials with the desired mechanical properties. The gelation process was monitored by SAOS following the evolution of *G*′ and *G*″. The crossover point between *G*′ and *G*″ was taken as indicative of the gelation time as it represents the transition from a liquid to a solid (gel). The initial formulation was prepared using 1.00 equiv of the N-substituted eight-membered bis-cyclic carbonate (8MCC), 0.60 equiv of PEG 3400, and 0.40 equiv of TAEA trifunctional amine. We first investigated the influence of the water content in the gelation process. With a water content of 50 wt %, gelation took 350 min to occur, while when the water content was increased to 70 wt %, the gelation took 750 min (Figure S3). Thus, as might be expected, the reaction was highly concentration-dependent such that higher water contents required longer reaction times. In fact, for high water contents (90 wt %), the crossover was not observed and no hydrogel was obtained even after 24 h (Figure S3). As expected, higher reactivity was also observed when the reaction was performed at higher temperatures (Figure S4). In addition, it was found that gels containing high water contents were not structurally stable in aqueous condition as the hydrolysis of the urethane groups occur (Figure S5).^27^ In contrast, gels with lower water contents were stable. The products of the hydrolysis have been analyzed by ^1^H NMR spectroscopy, and we found peaks that could be attributed to PEG as well as residues from amines and cyclic carbonate moieties. As such, the degradation of the hydrogels was attributed to the hydrolysis and decarboxylation of urethane groups as observed in the literature.

After optimizing the polymerization conditions (70 wt % water, rt), different ratios of PEG diamine and TAEA were investigated to obtain hydrogels with different properties and thermal responses (Table S1). As expected, changing the diamine/tris-amine ratio significantly affected the reaction kinetics (Figure S6). The higher cross-linker content translated into faster gelation times, ranging from 1780 to 200 min when the amount of TAEA was increased from 0.20 to 0.60 equiv (Table S1). This behavior can be ascribed to both an increase in the cross-linking points and a higher reactivity of the low-molecular-weight tris-amine in comparison to PEG diamine. In fact, when 0.80 equiv of TAEA were used, gelation was observed within seconds. At high cross-linking concentrations and low water contents (fast gelation conditions), the high reactivity of the TAEA can even lead to an inhomogeneous gel formation, as can be concluded by *G*′ values following gelation (see Figure S6). The mechanical properties of the obtained hydrogels were measured by DMTA in compression mode at a constant frequency of 1 Hz (Figure S7). All hydrogels showed a constant-plateau modulus in all samples, as expected for cross-linked networks, and an increased mechanical performance when the amount of cross-linker was increased. Hence, materials with tunable modulus between 1 × 10^5^ and 4 × 10^5^ Pa could be attained.

### Crystallization-Induced Gelation and 3D Printing Behavior

One of the most interesting characteristics of semicrystalline hydrogels is their good mechanical properties at room temperature together with their ability to flow during the printing process.^28^ To investigate the thermal responsivity of the NIPU hydrogels, we focused our study on a representative formulation (Table S1, entry 2). A similar effect was also observed for hydrogels containing different amounts of cross-linking points (Table S1, entries 1 and 3, Figure S8).

**Figure 2:**
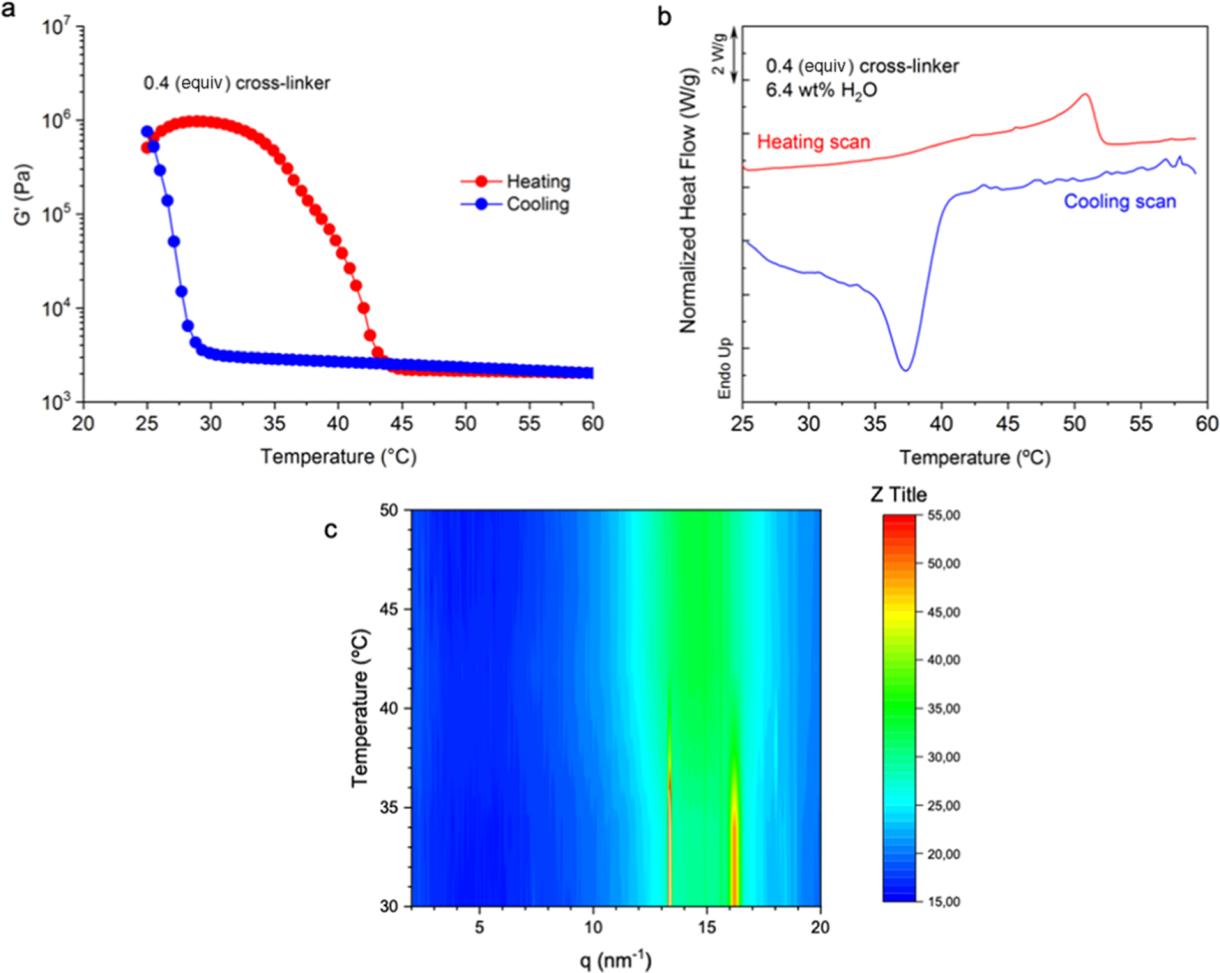
All of the analyses have been performed with hydrogels containing 0.4 equiv of cross-linker after adjusting the water content. (a) Temperature-resolved rheological testing was carried out under linear viscoelastic conditions to evaluate the change of the moduli as a function of temperature. (b) Temperature-resolved DSC testing was carried out to determine the melting/crystallization of entry 2 containing 6.5 wt % water. (c) *In situ* temperature-resolved grazing-incidence wide-angle X-ray scattering (GIWAXS) data.

First, we investigated the evolution of the materials’ rheological behavior during consecutive heating and cooling cycles. During the first two cycles, the hydrogels showed no response to temperature and displayed a low modulus (10^3^ Pa). We hypothesize that as the hydrogel had a large amount of water, the crystallizable regions were not able to properly crystallize as water was present between the crystallizable units (Figure S9). However, after removing 50 wt % water in the first two cycles, when cooling down the sample to 25 °C during the second cooling step, *G*′ slowly increased until a sharp increase in the modulus to 10^5^ Pa was observed around 27 °C (Figure 2a, blue). During the subsequent heating, a sharp decrease in the modulus was observed in the range of 35–45 °C (Figure 2a, red). Subsequent cooling–heating cycles generated the same response, with a small increase in the *G*′ value at low temperatures as a consequence of water evaporation. Although materials show melting–crystallization characteristics, they show a predominantly elastic behavior (*G*′ > *G*″) in the whole temperature range.

The observed behavior appeared to be related to the crystallization of PEG moieties in the hydrogel during the cooling process, which can increase its mechanical properties. We decided to confirm this hypothesis by further characterizing the sample containing 6.4 wt % water using differential scanning calorimetry (DSC). As expected, samples that contained low water contents showed an endothermic peak around 50 °C (Figure 2b, red). Similarly, cooling scans showed exothermic peaks, in the range of 37–30 °C (Figure 2b, blue). Both the endothermic and exothermic peaks can be ascribed to the melting and crystallization of PEG domains, respectively.^29,30^ To confirm that this change was ascribed to PEG crystalline domains, *in situ* X-ray diffraction analysis as a function of temperature was carried out in grazing incidence wide angle scattering (GIWAXS) configuration (Figure 2c). This experiment confirmed that the endothermic processes observed by DSC result from the melting of PEG crystals as diffraction peaks in the (120) and (032) planes from PEG crystals disappear between 35 and 38 °C, coinciding with the endothermic processes in DSC thermograms.

**Figure 3:**
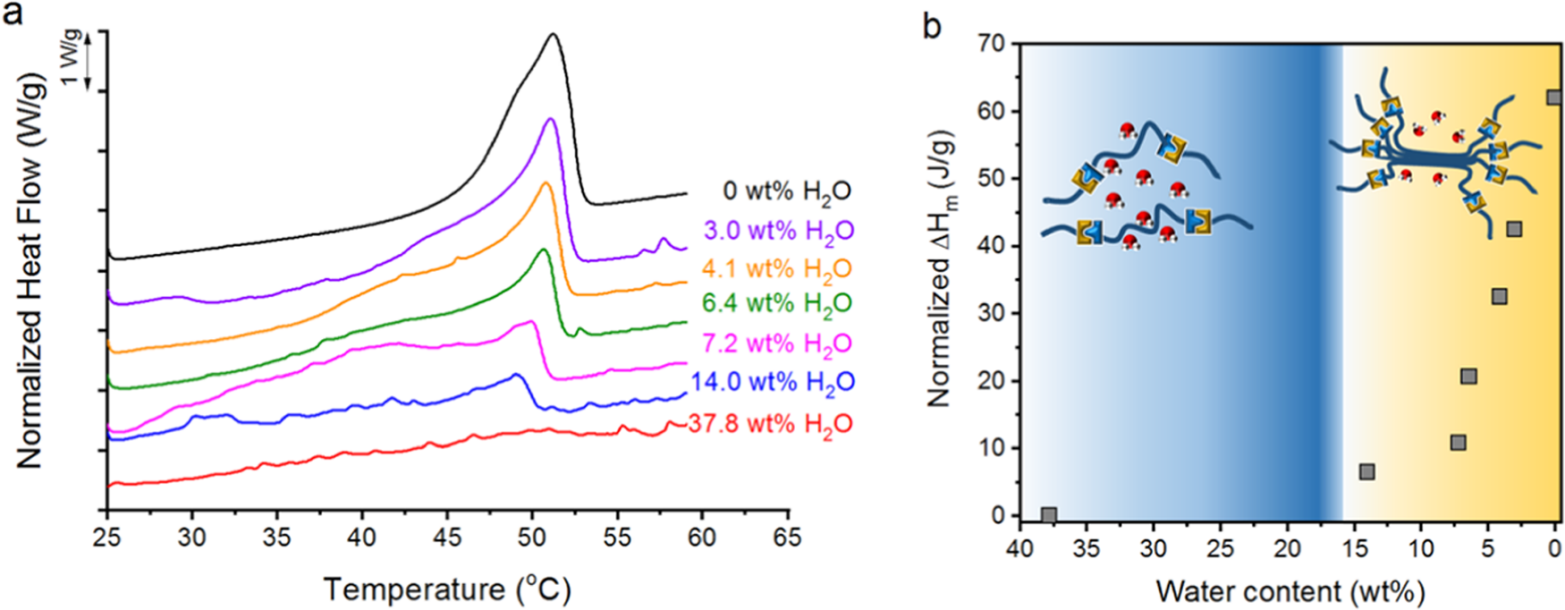
(a) DSC heating scans for the hydrogel with different water contents. (b) Evolution of melting enthalpy as a function of water content in the sample.

Furthermore, we conducted DSC analysis by carrying out consecutive heating/cooling scans and calculating the water content by mass loss after each cycle (see the Supporting Information and Figure 3a) to evaluate the effect of water on crystallization. Thus, a high water content (above 40 wt %) showed no remarkable thermal transition in the 25–60 °C range. However, as the water content reached 15 wt %, some changes in the thermal transition were observed (Figures 3a and S10). Similarly, the mechanical properties of the hydrogel change in this water content region, showing a sharp increase in the storage modulus as the water content is reduced (Figure S11). This effect is attributed to the high efficiency of crystalline domains to support the hydrogel structure as well as the low water content. Both rheology and DSC data suggest that a minimum amount of water is necessary to “solvate” PEG domains in the hydrogel. Below this critical amount, PEG chains start to crystallize and the melting and crystallization of these are responsible for the change in the mechanical properties. The synthetic flexibility of the proposed hydrogels allows the modification of different parameters to change not only the mechanical performance but also the water content needed to trigger the printing process. Thus, we synthesized similar gels using a PEG diamine with a molecular weight of 8000 g/mol. These gels present melting characteristics, both in rheological and DSC measurements (Figure S12). As shown, PEG 8000-based gels present the endothermic peak attributed to the melting of PEG domains. By plotting the normalized melting enthalpy against the hydrogel water content, it was possible to calculate the maximum water amount allowed for crystallization to still occur (15 wt%), and subsequently for the hydrogel to present crystallization (Figure 3b). These results highlighted the necessity of adjusting water content to allow the crystallization of PEG domains in hydrogels.

After confirming that the sharp decrease in the mechanical properties was related to the formation of microcrystals within the hydrogel at water contents below 15 wt%, it was also observed that an increase in temperature led to melting of the crystallized moieties and the material reverted to its initial mechanical properties. The critical water content value depends on the composition of the hydrogel. PEG 8000-based hydrogels, for instance, reflect the characteristic melting transition at higher water contents (27 wt%) (see Figure S12c).

**Figure 4:**
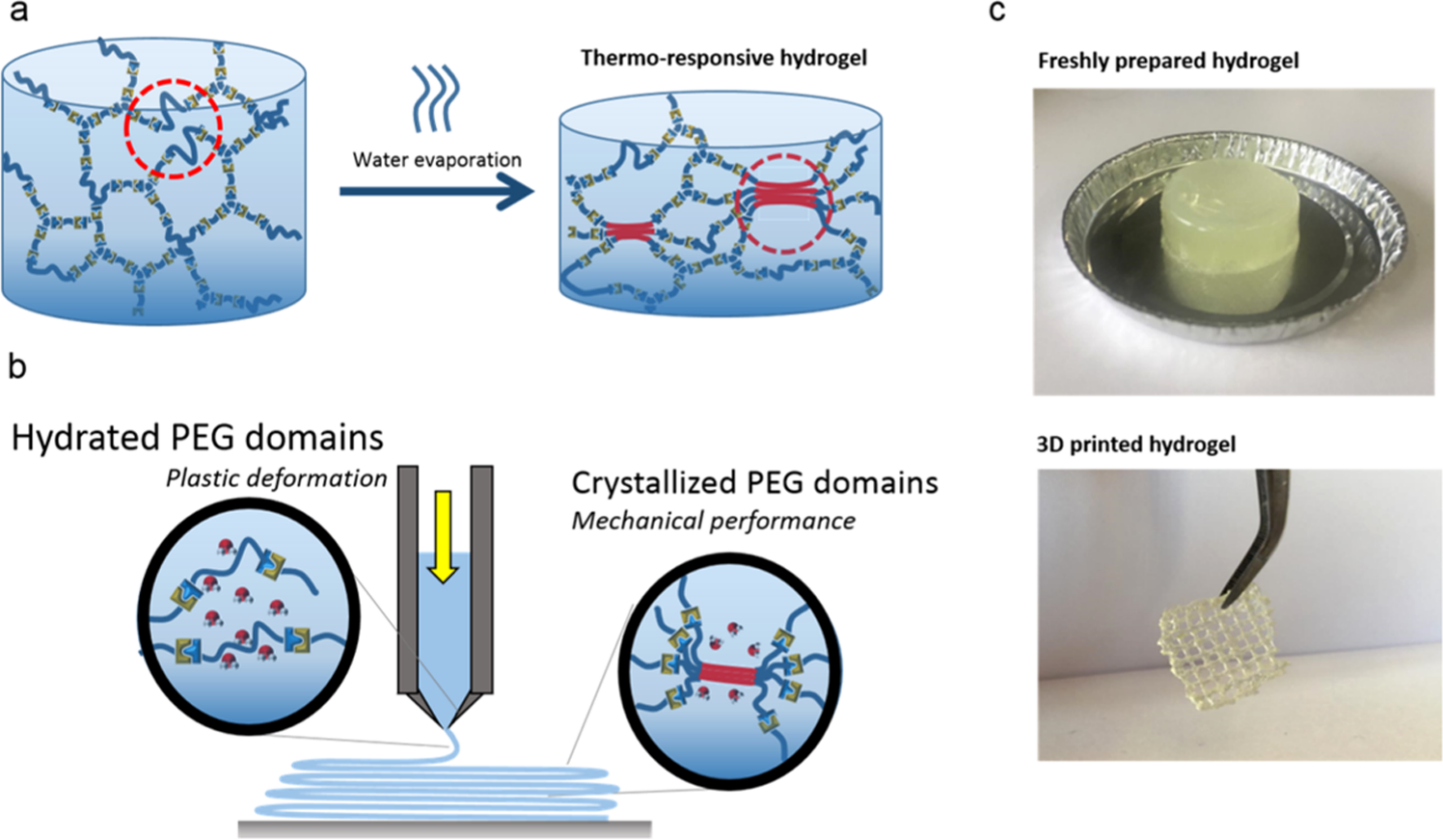
(a) Heat treatment performed to obtain temperature-responsive hydrogels. (b) Proposed printing mechanism of semicrystalline hydrogels based on the plastic deformation and recovery of the mechanical properties. (c) Three-dimensional printed scaffolds after the heat treatment (water content at 6.4 wt %) (c).

Taking advantage of the crystal melting process, we then investigated the suitability of these materials as inks for air-assisted extrusion 3D printing, after properly adjusting the water content to favor the abrupt change in the modulus (Figure 4a). As expected, the printing capabilities were strongly dependent on the crystallization behavior and, therefore, on the hydrogel water content. Thus, freshly prepared hydrogels were first heat-treated (30 min in the printing cartridge at 50 °C) to promote water evaporation inside the cartridge and to provide the thermo-responsive behavior. Under such conditions, the low moduli of the material at a high temperature (above the transition) allowed us to extrude the chemically cross-linked hydrogel, even though this material is not able to flow. We hypothesize that the low modulus of the material allows its plastic deformation and the extrusion by material slippage. To confirm this, viscosity measurements were performed above and below the transition temperature (Figure S13). Above the transition, the material shows the characteristic viscosity curve for a “plug flow”, with a slope of −1.^31,32^ Importantly, after extrusion and cooling, the gel was able to recover sufficient viscoelastic properties to maintain the printed shape after exiting from the nozzle (Figure 4c). Such crystallizable hydrogels were printed at 40 °C under 1.5 bar air pressure, resulting in a 4 mm/s printing speed (Figure 4c). In this case, materials were able to maintain the geometrical structure after printing.

**Figure 5:**
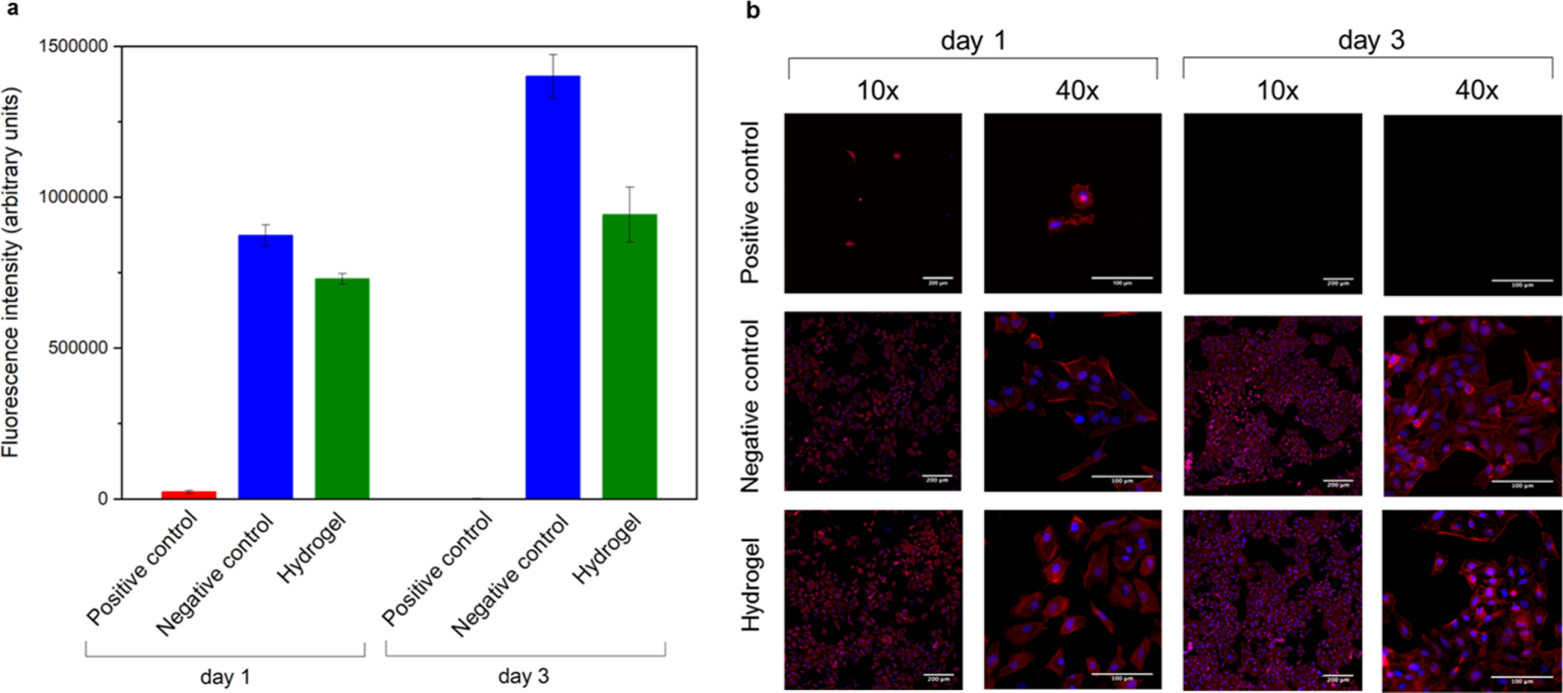
Metabolic activity (a) and fluorescence imaging (b) of HeLa cells exposed to positive control (*i.e.*, complete media + 10% DMSO), negative control (*i.e.*, complete media), or extracts of hydrogels. (Nuclei-DAPI: blue/actin filaments-rhodamine phalloidin: red).

To evaluate the potential of these materials for biomedical applications, their cytocompatibility was investigated. The hydrogels were not structurally stable in media for 24 h, making the direct seeding of the cells on top of them impossible. Hence, to evaluate cytotoxicity, HeLa cells were incubated for 3 days in media that had been incubated with the hydrogel for 24 h and 37 °C (Figure 5a). To observe cell morphology and analyze their spreading and proliferation, the cells were stained with rhodamine phalloidin (red) and DAPI (blue) (Figure 5b). In the presence of extracts of hydrogels, individual cells showing typical epithelial cell morphology were observed on day 1 and no differences were detected with respect to cells seeded in the presence of the negative control. On day 3, a higher number of cells were observed in both samples, proving the ability of this cell line to proliferate in the extract of the hydrogels. Contrarily, cells seeded in the presence of the positive control showed a round morphology on day 1, while no cells were observed on day 3.

Considering that the chemistry of the hydrogel may be altered during its incubation, we also analyzed the metabolic activity of cells in the presence of the hydrogel. For that, we incubated the hydrogel with cells with the aid of a transwell membrane to avoid direct contact of the hydrogel with cells (Figure S14a) and analyzed the metabolic activity after 1 day in culture. This incubation time is enough to allow the disassembly of the hydrogel structure. As observed in the optical micrographs (Figure S14b), the cells displayed a normal morphology in the presence of the hydrogel, whereas they were round-shaped in the positive control (complete medium + 10% DMSO). These results were further confirmed by the metabolic activity measurements, where cells in the presence of the hydrogel had similar metabolic activity with respect to the negative control (complete medium) (Figure S14c).

## Conclusions

We have shown how the crystallization process of poly(ethylene glycol) can be leveraged to print non-isocyanate poly(hydroxyurethane) hydrogels by inject printing. With careful design and as a consequence of the crystallization process, the storage modulus of the hydrogel can be tuned up to 3 orders of magnitude as a function of temperature. Thus, while the material is able to plastically deform at 45 °C, it rapidly recovers its mechanical performance upon cooling down to 25 °C. The capability of altering the physical properties of the hydrogels at different temperatures allowed the hydrogels to be 3D-printed *via* an extrusion printing method. Finally, cytocompatibility studies demonstrated that these materials are noncytotoxic and allow for cell spreading and proliferation. We expect that the crystallization-induced gelation will enable us to expand the number of 3D-printable hydrogel inks that may serve as interesting materials for tissue engineering scaffolds and other biomaterial applications as it provides a unique platform to 3D-print pre-formed biocompatible hydrogels with suitable mechanical properties avoiding the use of postfunctionalization treatment.

## Supporting Information Available

Complementary procedures and ^1^H and ^13^C NMR spectra (PDF)
