# Supplementary material for: Crystallization-Induced Gelling as a Method to 4D Print Low-Water-Content Non-isocyanate Polyurethane Hydrogels
Source: Chem Mater. 2021 Sep 14;33(18):7194–202. doi: 10.1021/acs.chemmater.1c00913 (PMC8482781; doi:10.1021/acs.chemmater.1c00913)
Supplement: Supplementary file 1 — cm1c00913_si_001.pdf [file cm1c00913_si_001.pdf]

Supporting information

Crystallization-Induced Gelling as a method to 4D print low water content non-isocyanate polyurethane hydrogels

Noé Fanjul-Mosteirín,[1,2,3] Robert Aguirresarobe,[2] Naroa Sadaba,[4] Aitor Larrañaga,[4] Edurne Marin,[4] Jaime Martin,[2,5] Nicolas Ramos-Gomez,[2] Maria C. Arno,[1] Haritz Sardon,[,2] Andrew P. Dove*[,1]*

[1] School of Chemistry, University of Birmingham, Edgbaston, Birmingham, B15 2TT, UK.

[2] Department of Chemistry, University of Warwick, Gibbet Hill Road, Coventry, CV4 7AL, UK.

[3] POLYMAT, University of the Basque Country UPV/EHU, Joxe Mari Korta Center, Avda Tolosa 72, 20018 Donostia-San Sebastian, Spain.

[4] Department of Mining-Metallurgy Engineering and Materials Science, POLYMAT, University of the Basque Country UPV/EHU, School of Engineering, Alameda de Urquijo s/n, 48013 Bilbao, Spain.

[5] Universidade da Coruña, Grupo de Polímeros, Departamento de Física e Ciencias da Terra, Centro de Investigacións Tecnolóxicas (CIT), Esteiro, Ferrol, 15471 Spain

Table of contents

reScheme S1

Synthesis of 6,6'-(ethane-1,2-diyl)bis(1,3,6-dioxazocan-2-one)

Synthesis of 4,4'-(oxybis(methylene))bis(1,3-dioxolan-2-one)

Synthesis of poly(hydroxyurethane) linear polymers

Synthesis of poly(hydroxyurethane) hydrogels

References

Materials

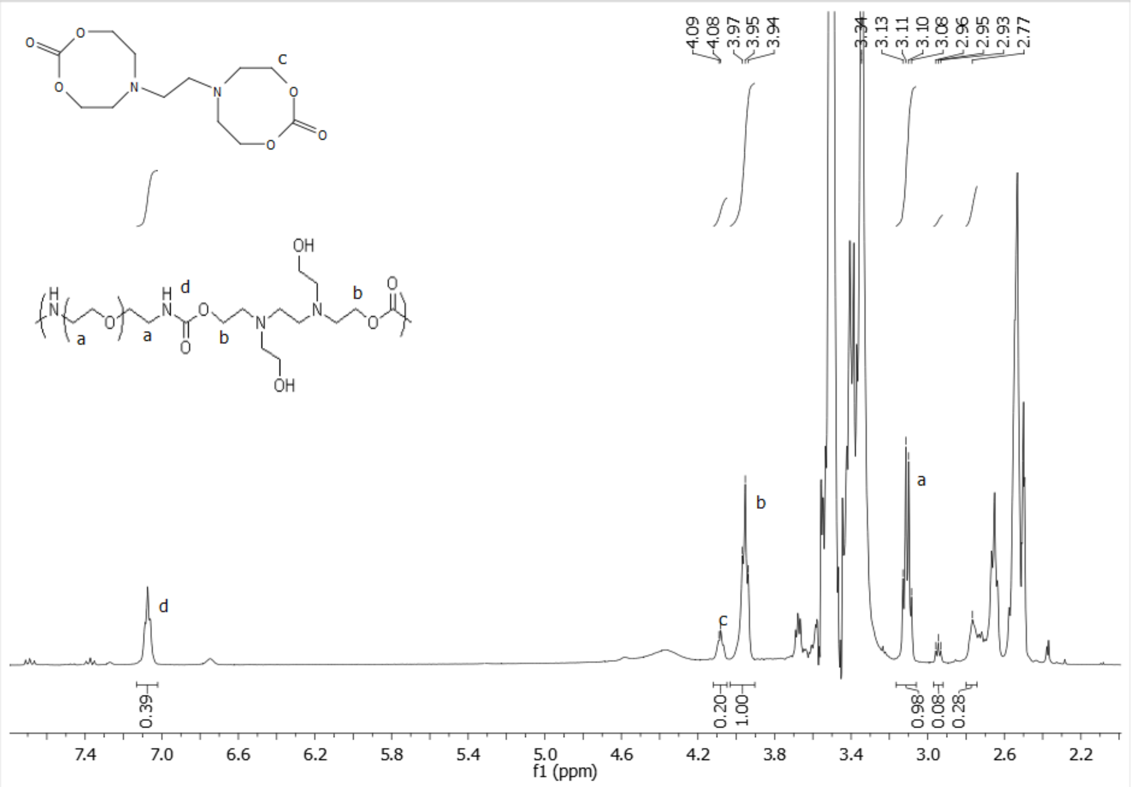

Figure S1. ^{1}H NMR spectrum of *in situ* reaction between 8MCC and PEG-1000 at R.T overnight. The reaction conversion was calculated with the following equation: $\%\ conversion = \frac{b}{(b+\frac{c}{2})}$. The final conversion was 91%.

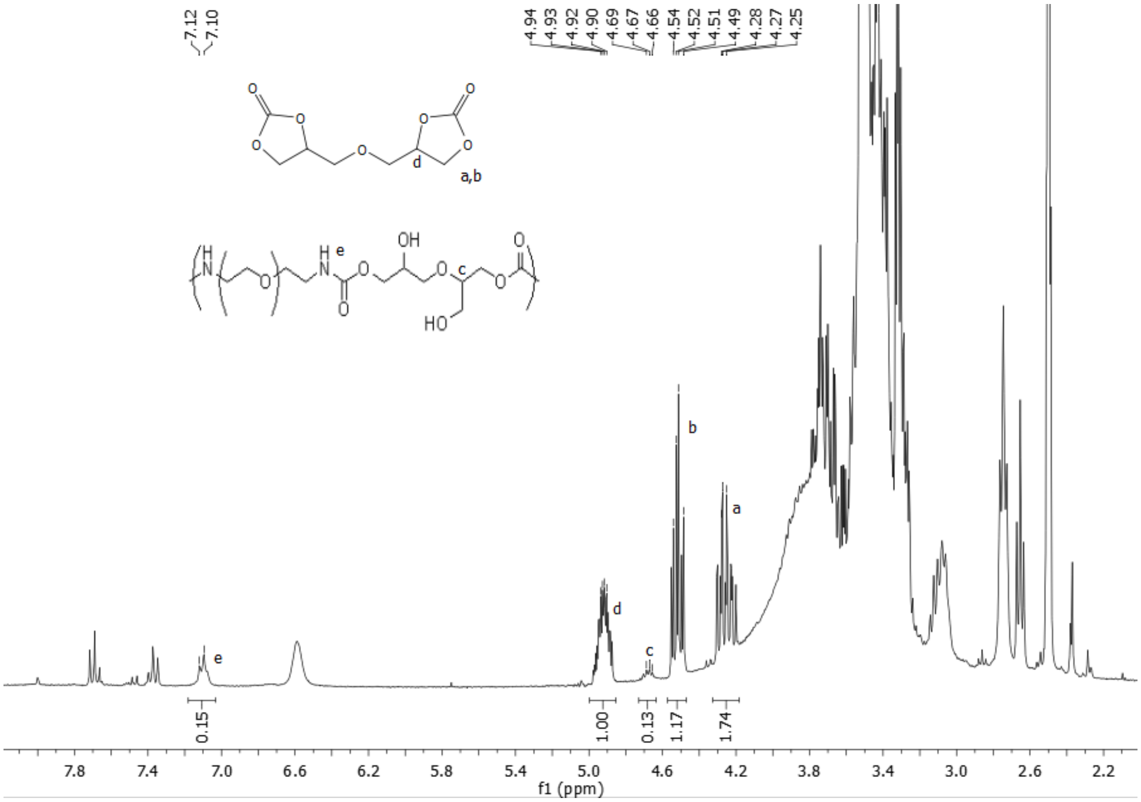

Figure S2. ^{1}H NMR spectrum of *in situ* reaction between bis glycerol carbonate and PEG-1000 at R.T. The conversion was calculated as follows: $\%\ conversion = \frac{a}{(a+b)}$. In this case, the overnight conversion was 12%.

Synthesis of poly(hydroxyurethane) hydrogels.

In a 5 mL vial, a stock solution of TAEA 0.2 M was added, then water was added to obtain a final volume of 400 μL. Then PEG diamine was added to the previous mixture and the vial was stirred in a vortex until total dissolution of the PEG diamine. Subsequently 6,6'-(ethane-1,2-diyl)bis(1,3,6-dioxazocan-2-one), was added to the mixture in a vortex until was dissolved. Mixture was left overnight at room temperature without further stirring and a gel was obtained.

Table S1. Synthesis of different PHU-based semicrsytalline hydrogels using different ratios of PEG diamine and TAEA.

**Entry**	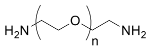 M_w = 3,400 **(eq)**	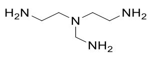 **(eq)*a***	Water content **(wt%)**	Temperature **(ºC)**	Gelation time **(min)**
1	0.60	0.40	53	25	350
2	0.60	0.40	70	25	690
3	0.60	0.40	90	25	No gel
4	0.60	0.40	70	37	625
5	0.60	0.40	70	15	765
6	0.60	0.40	70	4	> 2500
7	0.80	0.20	70	25	1780
8	0.40	0.60	70	25	200
9	0.20	0.80	70	25	< 1[a]
10	0.60	0.40	70	25	-

1 equivalent of N-substituted eight-membered bis-cyclic carbonates and 2/3 mmol of TAEA respect with eight-membered cyclic carbonate were used in all cases. [a] Entry 9 was not able to be measured by rheometer as the gelation occurred in less than 1 minute. [b] Entry 10 was synthesized using a PEG diamine of M_w=8000 g/mol.

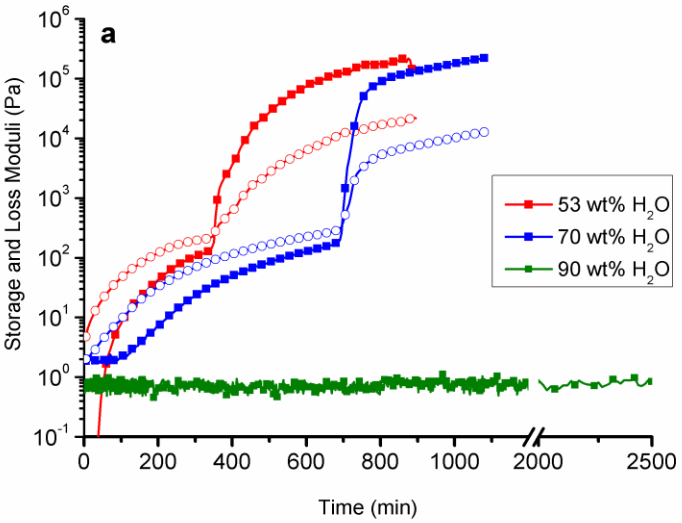

Figure S3. Evolution of the reaction kinetics depending on the water content for an 8MCC/PEG 3,400/TAEA (1/0.6/0.27 equivalents) formulation.

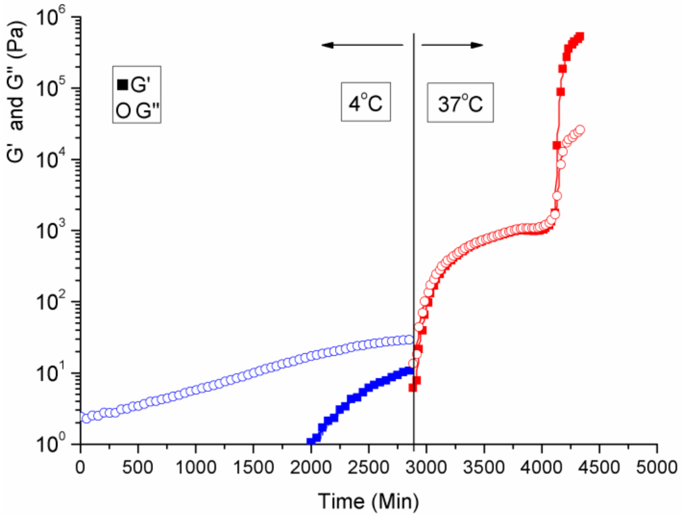

Figure S4. Modulus evolution at different temperatures for an 8MCC/PEG 3,400/TAEA (1/0.6/0.27 equivalents) formulation.

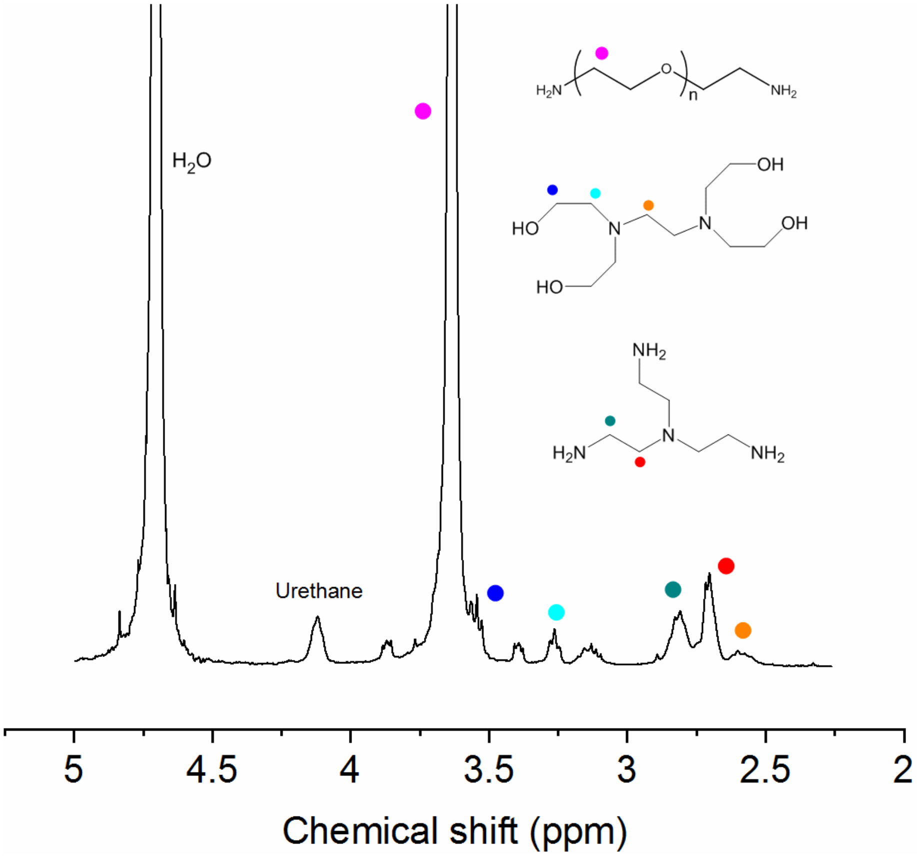

Figure S5. Representative ^{1}H-NMR spectrum for the degraded hydrogel. The spectrum was performed by mixing the degraded residue with D_2O.

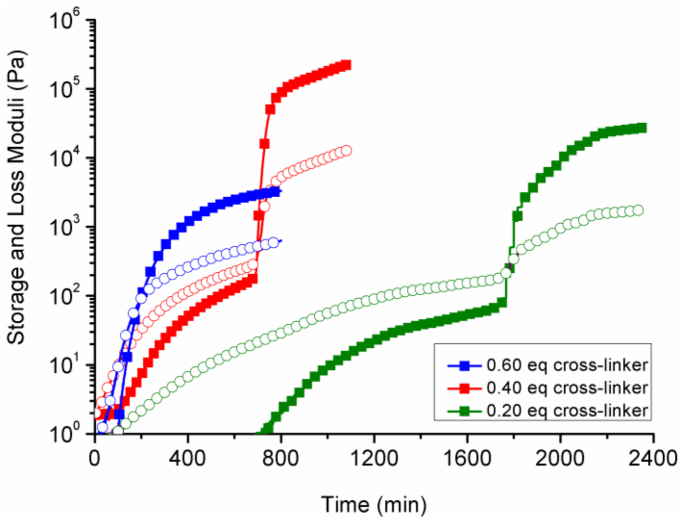

Figure S6. Evolution of the cross-linking reaction for samples containing different PEG 3400/TAEA ratios and 70 wt% of water.

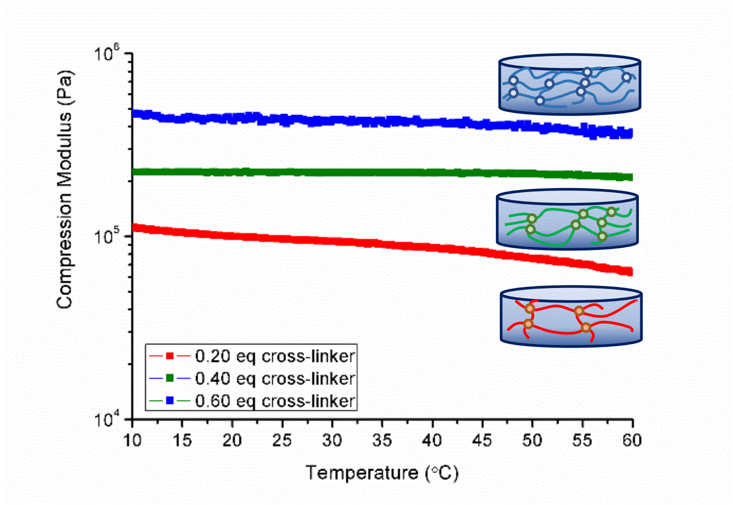

Figure S7. Thermomechanical properties of hydrogels containing different amounts of crosslinker.

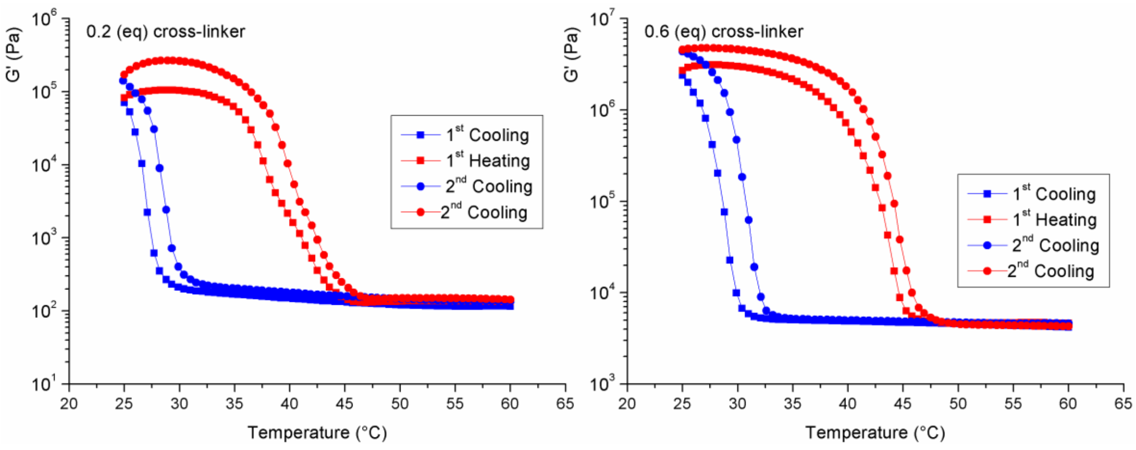

Figure S8. Temperature sweep experiment for formulations containing 0.2 and 0.6 equiva07. 0lents of cross-linker (Entries 7 and 8).

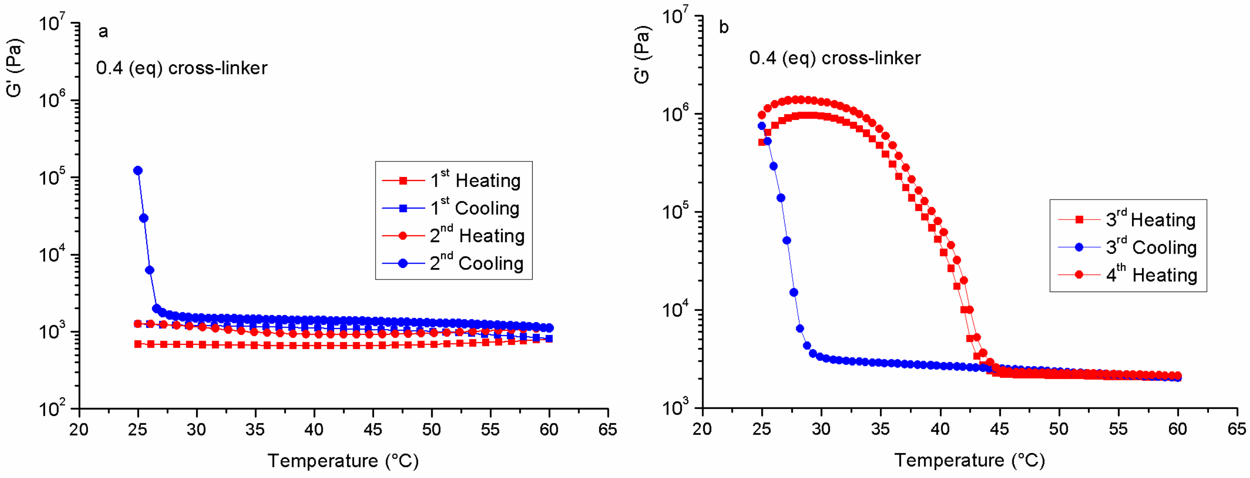

Figure S9. Temperature sweep experiment for formulations containing 0.4 equivalents of cross-linker before a) and after b) adjusting the water content. As expected when the water content is too high no change in the modulus is observed.

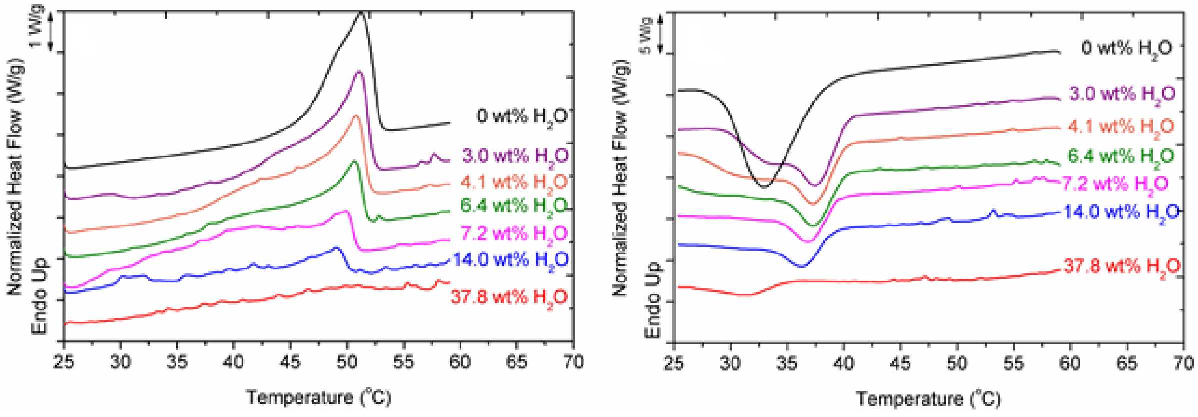

Figure S10. Thermal properties of the hydrogel containing 0.4 equiv. of crosslinker as a function of water content. a) DSC thermograms (heating scan) for the hydrogel with different water contents and b) DSC thermograms (cooling scan) for the hydrogel with different water content

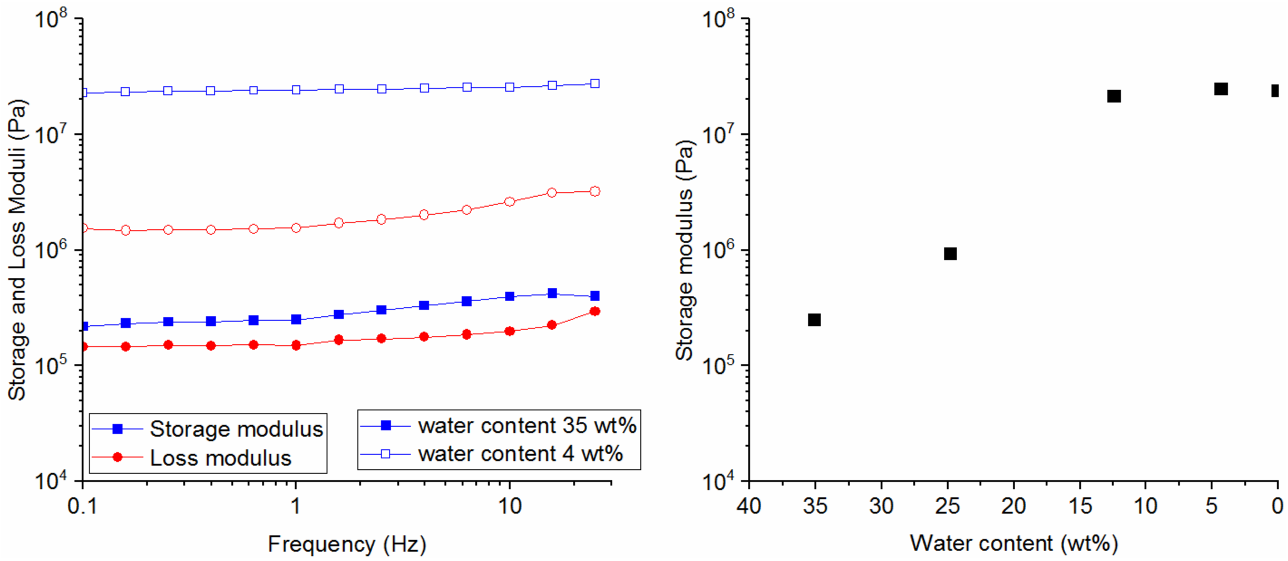

Figure S11. A) Representative DMTA results for hydrogels containing 0.4 equiv. of crosslinker and different amounts of water. B) Evolution of the Storage modulus of the hydrogel as a function of the water content.

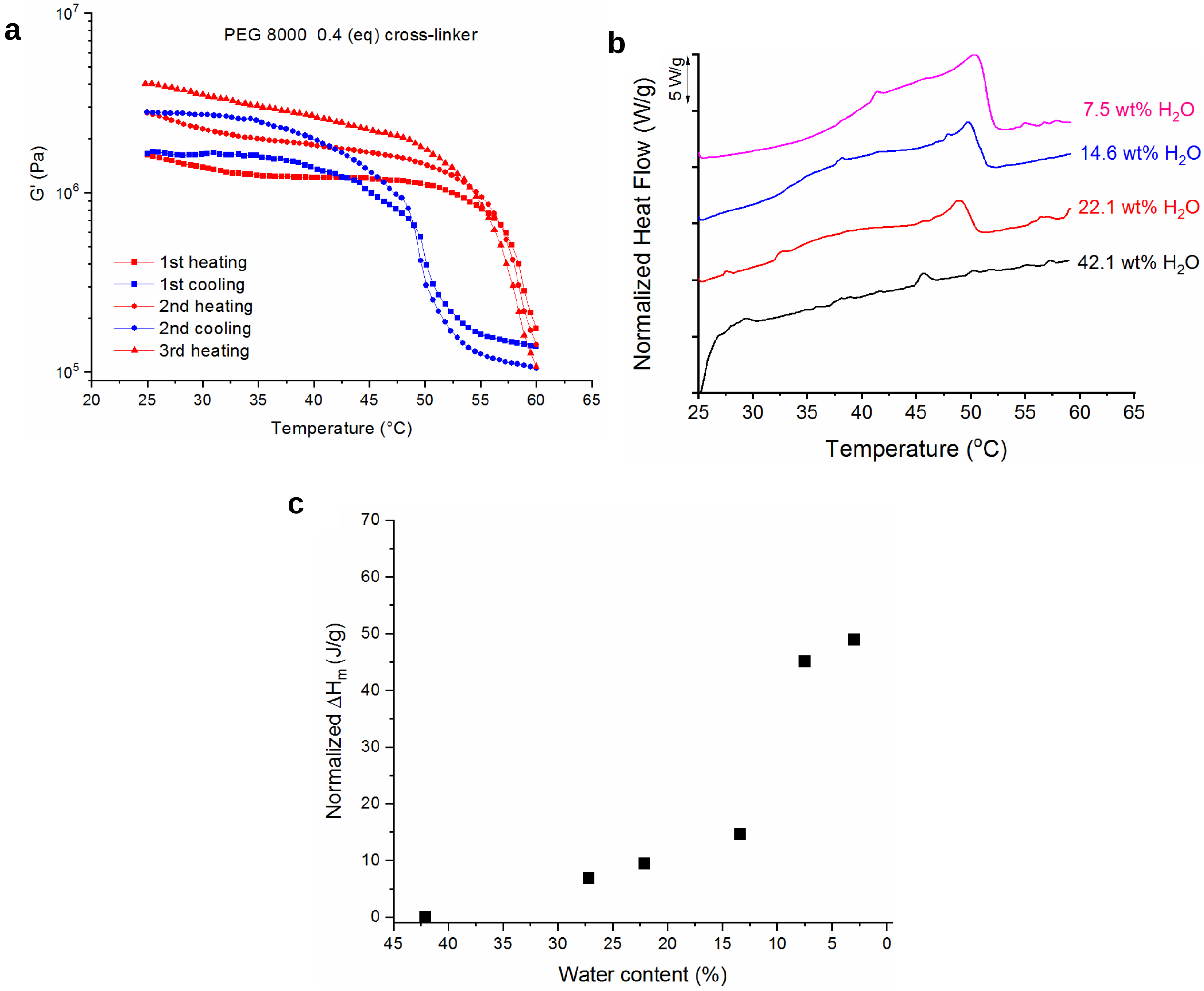

Figure S12. A) Temperature sweep experiment for formulations synthesized PEG8000 and containing 0.4 equivalents of cross-linker before (Entry 10). b) Representative DSC traces for the synthesized hydrogel and c) the evolution of the melting enthalpy with the water content.

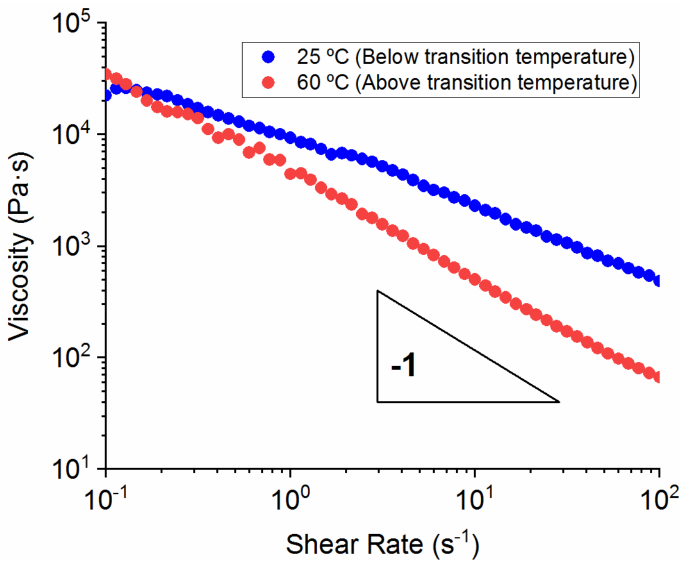

Figure S13. Viscosity curves for the hydrogels below and above the transition temperature. At 60ºC it can be observed a characteristic slope of -1, representative of the total slippage ("plug flow") of the hydrogel.

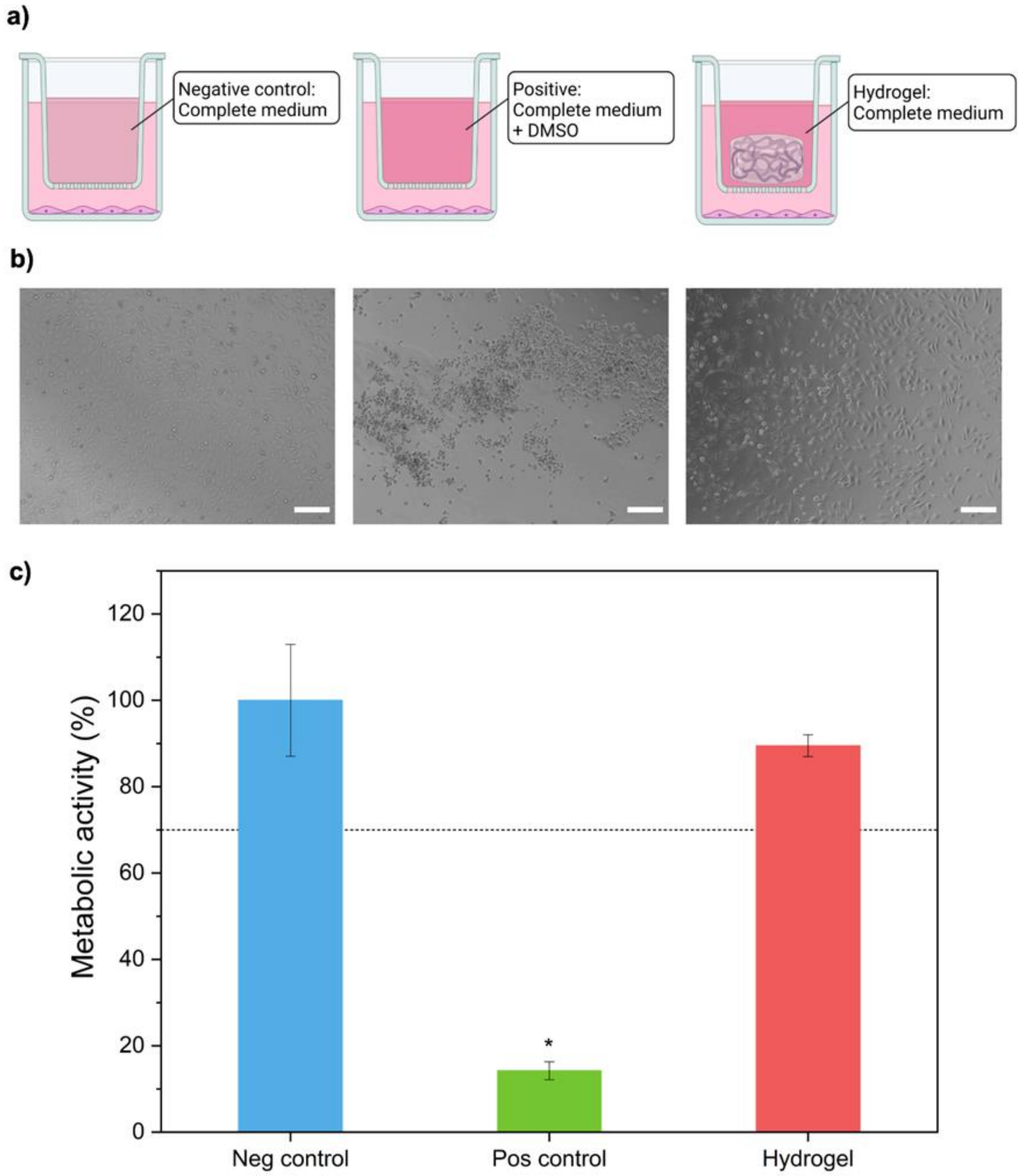

Figure S14. a) Schematic representation of the experimental setup to allow the incubation of the hydrogel with cells. b) Optical micrographs of HeLa cells after 1 day in culture with the negative control (left), positive control (middle) and hydrogel (right). Scale bar: 200 μm. c) Metabolic activity of HeLa cells after 1 day in culture. Asterisks indicate significant differences ($p<0.05$) with respect to the negative control.

REFERENCES

(1) Tryznowski, M.; Świderska, A.; Żołek-Tryznowska, Z.; Gołofit, T.; Parzuchowski, P. G., Facile Route to Multigram Synthesis of Environmentally Friendly Non-Isocyanate Polyurethanes. *Polymer* **2015,** *80*, 228-236.
